# A Pipeline to Understand Emerging Illness Via Social Media Data Analysis: Case Study on Breast Implant Illness

**DOI:** 10.2196/29768

**Published:** 2021-11-29

**Authors:** Vishal Dey, Peter Krasniak, Minh Nguyen, Clara Lee, Xia Ning

**Affiliations:** 1 Department of Computer Science and Engineering The Ohio State University Columbus, OH United States; 2 Department of Biomedical Informatics The Ohio State University Columbus, OH United States; 3 Translational Data Analytics Institute The Ohio State University Columbus, OH United States

**Keywords:** breast implant illness, social media, natural language processing, topic modeling

## Abstract

**Background:**

A new illness can come to public attention through social media before it is medically defined, formally documented, or systematically studied. One example is a condition known as breast implant illness (BII), which has been extensively discussed on social media, although it is vaguely defined in the medical literature.

**Objective:**

The objective of this study is to construct a data analysis pipeline to understand emerging illnesses using social media data and to apply the pipeline to understand the key attributes of BII.

**Methods:**

We constructed a pipeline of social media data analysis using natural language processing and topic modeling. Mentions related to signs, symptoms, diseases, disorders, and medical procedures were extracted from social media data using the clinical Text Analysis and Knowledge Extraction System. We mapped the mentions to standard medical concepts and then summarized these mapped concepts as topics using latent Dirichlet allocation. Finally, we applied this pipeline to understand BII from several BII-dedicated social media sites.

**Results:**

Our pipeline identified topics related to toxicity, cancer, and mental health issues that were highly associated with BII. Our pipeline also showed that cancers, autoimmune disorders, and mental health problems were emerging concerns associated with breast implants, based on social media discussions. Furthermore, the pipeline identified mentions such as rupture, infection, pain, and fatigue as common self-reported issues among the public, as well as concerns about toxicity from silicone implants.

**Conclusions:**

Our study could inspire future studies on the suggested symptoms and factors of BII. Our study provides the first analysis and derived knowledge of BII from social media using natural language processing techniques and demonstrates the potential of using social media information to better understand similar emerging illnesses.

## Introduction

### Background

The ubiquity of social media has resulted in early descriptions of new and evolving diseases on social media platforms before they can be systematically studied [1-7], particularly during the era of the medical internet [8-14]. Social media users increasingly turn to platforms such as Twitter (Twitter Inc), Facebook (Facebook Inc), and YouTube (Google LLC) to share personal experiences, including diseases and illnesses they have experienced, or to seek support and resources, such as health and medical resources. Recent studies have shown the potential of social media in the detection of mental illness and depression [15-17] and in the early detection of food-borne illnesses [18-20] and other infectious diseases [2,21-24]. Furthermore, several studies have demonstrated social media as an effective tool to disseminate information regarding symptoms, personal well-being, and public health resources during multiple influenza outbreaks [25-28]. During the early stages of COVID-19, studies [4,29,30] analyzed posts on Sina Weibo (Weibo Corporation)—a major Chinese microblogging site—to characterize patient symptoms and public concerns in multiple provinces of China. From the analysis of Weibo (Weibo Corporation) posts, Huang et al [30] concluded that most of the affected patients were older persons, with fever as the most common symptom. These studies demonstrate that public social media data can be leveraged to better understand emerging illnesses and to accommodate prompt responses.

One new illness we studied in this manuscript was breast implant illness (BII). Breast implants have gained popularity over the last 20 years [31]. During this period, more than 400,000 women have undergone breast augmentation or postmastectomy surgeries every year in the United States [32]. There was a 4% increase in the number of breast augmentation procedures between 2017 and 2018, and a 6% increase in breast implant removal procedures occurred over the same period [32]. Concerns about the safety of breast implants have also arisen [33-38] and persisted [39-45]. However, although a causal link between breast implants and systemic diseases has not been definitively shown, a phenomenon called *breast implant illness*, which attributes systemic symptoms to breast implants, has emerged [46]. Unlike other new medical illnesses, however, BII has been reported minimally in the medical literature, being primarily limited to social media [11,47-50]. For example, a recent analysis [49] demonstrated increasing public interest in BII based on Twitter and Google Trends data from February 2018 to February 2019. To summarize the key symptoms, diseases, and disorders defining BII, several cohort studies [51,52] have analyzed patient-reported outcomes before and after breast explant surgeries. These studies showed some potential relationships between explant surgeries and the improvement of specific symptoms in the patient population. Unfortunately, these studies were not definitive because of their limited study design secondary to their lack of control groups, data collection bias, and lack of randomization. The lack of medical knowledge about BII makes it difficult to define the condition, and therefore, it is nearly impossible to conduct rigorous epidemiological or clinical studies. BII is just one disease process for which the lack of medical knowledge is apparent, but there are many other new illnesses for which this is the case. Any initial knowledge that is supported by sufficient social media data would be meaningful as a reference for formal studies in the future, and thus, the techniques to discover such knowledge are highly required.

### Objectives

To identify and summarize the key attributes of a new illness, in this study, we constructed a data analysis pipeline for the social media data analysis of BII. The pipeline incorporated natural language processing (NLP) and topic modeling methods. Our primary objective is to derive novel knowledge about BII, a medical condition that has not yet been systematically studied and defined in the medical literature, by constructing a data analysis pipeline and applying the pipeline to social media data. As medical knowledge and literature on BII have not been established and the related concepts are not well defined or accepted, using social media data to understand emerging issues could be a meaningful starting point. We applied this pipeline to better understand the symptoms and signs associated with BII. To the best of our knowledge, this study is the first to use social media data to derive the knowledge of BII from social media. This demonstrates the potential of using social media information to better understand the conditions that have primarily been reported on social media. It also establishes the effectiveness of our pipeline and its potential application to understand other new illnesses. In the following discussion, we have described our analysis pipeline in the context of BII. However, our pipeline is not specific to BII and is applicable to other illnesses as well.

## Methods

### Data

We collected and used data from select social media websites. These websites were selected because they were dedicated to BII discussions and information and were focused on user groups with interest in BII. Often, dedicated social media websites (eg, forums and Twitter pages) are available for a particular illness or disease. For example, some dedicated websites [53-55] contain the stories and experiences of patients fighting different cancers, some [56,57] contain posts and stories of users experiencing chronic pain and illness, and others [58-60] contain stories and experiences from COVID-19 survivors. The social media sources used in our study were as follows:

- BII [61]: This was a dedicated public website with articles on BII-related topics and offered resources related to implant and explant procedures, etc. This website also allowed individuals to post their experiences and concerns about breast implants and related health issues. We extracted individual posts from the website (up to May 10, 2019), and the resulting data set was referred to as BIIweb.
- Healing BII [62]: This website contained information on postimplant disorders, postexplant healing, breast implant safety, etc. The discussion board of this website had multiple posts and comments on symptoms, signs, etc, which are experienced by individuals with a breast implant or by those who have undergone an explant. The data set extracted from the discussion board of this website (up to May 10, 2019) was referred to as HealingBII.
- Instagram posts about BII [63]: This website contained a collection of publicly available Instagram posts that used *breastimplantillness* as a hashtag. We extracted the associated texts for each Instagram post with a timestamp between January 10, 2012, and September 4, 2019. The data set extracted from this site was referred to as IG-BII.

All the comments and posts from the 3 websites were included in the corresponding data sets. Table 1 presents a summary of the social media data collected. The BIIweb data set had only 187 posts (where each post on average has 129 words, SD 124) but these were larger (larger average length of posts in words) on average than those in the other 2 data sets. HealingBII was the second largest data set, with 1920 posts, each with 85 words on average (l_avg_) (SD 107). IG-BII was the largest data set, with 28,987 posts and 123 words per post on average (SD 113).

**Table 1:** Statistical summary of social media data analyzed.

Data set	Posts^a^ (n=31,094), n (%)	l_max_^b^	l_min_^c^	l_avg_^d^, mean (SD)	Words^e^, n (%)
BIIweb	187 (0.6)	669	3	129 (124)	24,191 (0.64)
HealingBII	1920 (6.17)	1330	1	85 (107)	165,090 (4.38)
IG-BII	28,987 (93.22)	515	1	123 (113)	3,581,081 (94.98)

^a^Posts: the number of posts and comments in the respective data sets.

^b^l_max_: the minimum length of a post in words.

^c^l_min_: the maximum length of a post in words.

^d^l_avg_: the average length of posts in words.

^e^Words: the total number of words in the respective data sets.

### The Pipeline

#### Overview

**Figure 1:**
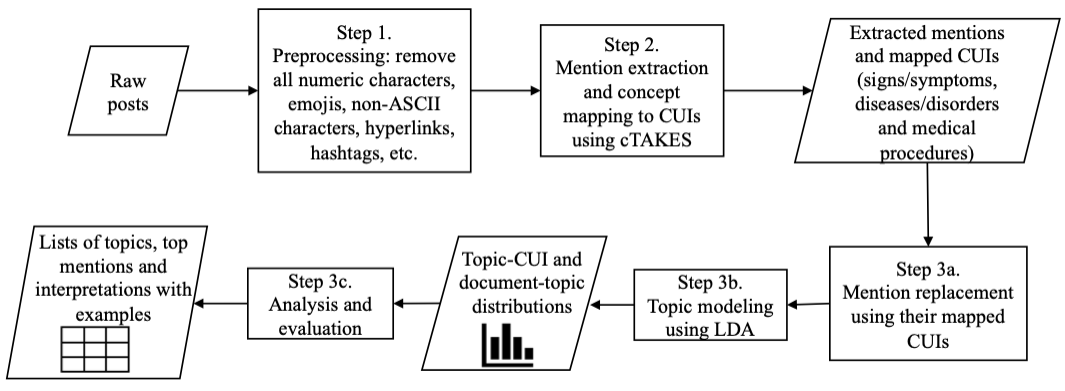
Pipeline for breast implant illness social media analysis. ASCII: American standard code for information interchange; CUI: concept unique identifier; LDA: latent Dirichlet allocation; cTAKES: clinical Text Analysis and Knowledge Extraction System.

Figure 1 shows an overview of the pipeline. We extracted major topics of interest primarily related to symptoms, diseases, and medical procedures from our data sets through the following 3 steps. Each of the steps will be discussed in detail later. The first step involved data preprocessing. We removed all stop words, numeric characters, hyperlinks, hashtags, etc, and converted the remaining characters into lowercase. The second step was of mention extraction and concept mapping. We extracted mentions related to signs, symptoms, diseases, disorders, and medical procedures using the clinical Text Analysis and Knowledge Extraction System (cTAKES) [64]. The extracted mentions were further mapped to standard medical concepts represented by concept unique identifiers (CUIs) in the unified medical language system (UMLS) [65] ontology. The third step involved topic modeling. We summarized the mapped concepts to topics using latent Dirichlet allocation (LDA) [66]. LDA is a probabilistic generative model for topic modeling. It represents each document as a mixture of latent topics, where each topic is modeled as a distribution over words. This modeling consisted of 3 stages: (1) mention replacement, (2) topic modeling using LDA, and (3) analysis and evaluation. In mention replacement, we replaced each extracted mention in the posts with its mapped CUIs and discarded all other words in the posts. We have discussed this step in more detail in the section *Topic modeling*. Then, in topic modeling using LDA, given the corpus of mapped CUIs, LDA generates document-topic and topics-CUI probability distributions. We have discussed this step in more detail in the section *Topic modeling*. Finally, during our analysis and evaluation, we further analyzed these distributions to derive a list of topics using the most representative mentions and summarized the extracted mentions for each data set. We have discussed this step in more detail in the section *Results: LDA topics*.

#### Data Preprocessing

We used the Natural Language Toolkit tokenizer [67] to tokenize the raw text for each data set. Out of the obtained tokens, we removed the stop-words (most frequently occurring, function words such as conjunctions, prepositions, determiners, etc) using the Natural Language Toolkit English stop-words list. As stop-words carried little or no information on our topics of interest in BII, they could be safely removed, as is typically done in NLP. We also removed all the numeric characters, emojis, non–American Standard Code for Information Interchange (ASCII) characters, hyperlinks, hashtags, and Instagram handles using regular expression matching and converted all the remaining tokens into lower cases to unify different cases for downstream processing.

#### Mention Extraction and Concept Mapping

Mention extraction refers to the extraction of words or phrases that convey a medical concept. We used the cTAKES tool for mention extraction. The cTAKES tool is an open-source NLP tool for clinical information extraction from unstructured clinical texts. cTAKES extracts mentions (ie, words or phrases that convey a medical concept) from posts and maps these mentions to standard medical concepts. In doing so, it also categorizes each extracted mention into one of 5 cTAKES categories: sign, symptom, disease, disorder, medication, procedure, and anatomy; that is, while cTAKES extracts mentions, it also automatically classifies the mentions into one of the 5 categories. For example, in the sentence “Over the years, my tinnitus has become worse to almost debilitating levels,” cTAKES extracts *tinnitus* as a mention of sign and symptom category. Below, we discuss how to configure the cTAKES in detail.

We used the fast-dictionary-lookup annotator in cTAKES to extract mentions from the processed data. This annotator identifies and extracts mentions in texts and normalizes them into CUIs in the UMLS standard medical ontology. This normalization of extracted mentions into CUIs is referred to as concept mapping. Each CUI in the UMLS ontology uniquely identifies a medical concept. Hence, we represented extracted mentions using the standard medical concepts of CUIs that cTAKES maps the mentions to. We configured the annotator to use an exact string match and to use the all-term-persistence property. Thus, the annotator could retain all terms, irrespective of the semantic properties of each term. For example, for the phrase *back pain*, the annotator would annotate the generic term *pain* as well as the precise term *back pain*. We chose to use the all-term-persistence property to retain maximum information with respect to precise and generic medical concepts. Finally, the annotator stored the generated annotations in XML Metadata Interchange (XMI) files.

**Figure 2:**
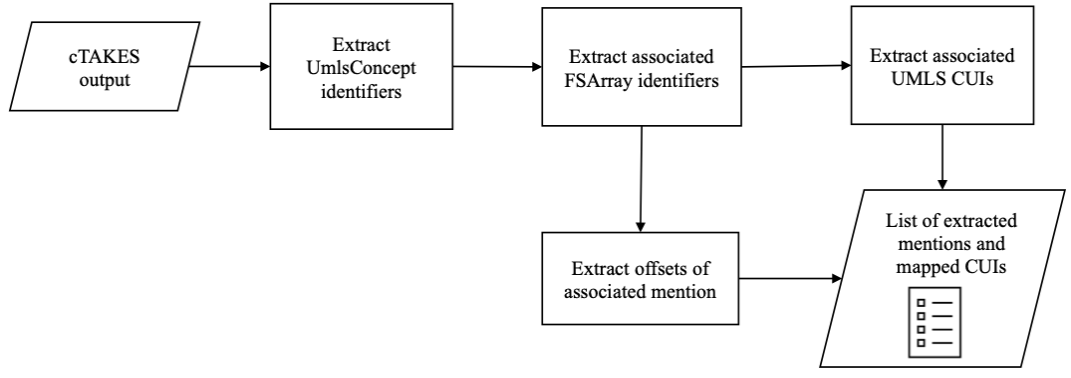
Pipeline for obtaining annotations out of Clinical text analysis and knowledge extraction system. cTAKES: clinical Text Analysis and Knowledge Extraction System; CUI: concept unique identifier; UMLS: unified medical language system.

To obtain the annotations in a human-readable format from the XMI files, we performed the following steps (Figure 2). We used a custom interpreter to process the XMI files produced by cTAKES and to obtain mappings between mentions and CUIs from cTAKES. We first searched for *UmlsConcept* XML identifiers in the XMI files, where each *UmlsConcept* XML identifier is generally grouped under the *FSArray*, and each *FSArray* is associated with a single ontology concept and the category of the concept. Each concept is assigned one category out of 5 cTAKES categories: sign, symptom, disease, disorder, medication, procedure, and anatomy. Each ontology concept is further associated with a UMLS CUI and an *ontologyConceptArr* identifier. It must be noted that a mention can be mapped to multiple CUIs. For example, the mention *allergic reaction* is categorized as sign and symptom but mapped to 2 different CUIs: *C1527304* and *C0020517*. Then, we extracted the ontology concepts that describe any of these categories: diseases, disorders, signs, symptoms, and medical procedures. Finally, we used the *begin* and *end* markers associated with each *ontologyConceptArr* identifier to obtain the position of the annotated mention in the input post. In this work, we were only interested in the first 3 categories (ie, sign, symptom, disease, disorder, and procedure) to understand BII-related issues. Hence, we only used the mentions categorized into either of these 3 categories.

#### Topic Modeling

To conduct topic modeling, we processed the posts as follows: we substituted each mention in the posts with its mapped CUIs and discarded all other words in the posts, which were considered as nonmedical concepts by cTAKES or were not among the 3 categories of interest. If a mention was mapped to multiple CUIs, we replaced it with multiple CUIs. If multiple mentions were mapped to the same CUI, we replaced all such mentions with the CUI. In this way, each post was represented as a bag-of-CUI, instead of a collection of mentions, as the input to the topic modeling and our vocabulary consisted of CUIs. Upon topic modeling, we interpreted the topic-CUI distribution to derive the topics.

We used LDA [66] to learn the topic distributions of each post and the CUI distributions of each topic. LDA is a generative probabilistic model for modeling topics within a document corpus. LDA models each document in the corpus as a mixture of latent topics, where each topic is modeled as a distribution over words in all documents. LDA derives the optimal distributions by maximizing the likelihood of observing the corpus, following perspective distributions. A brief description of LDA is provided in Multimedia Appendix 1 [66]. In our experiments, a bag-of-CUIs generated as described above was used as a document in LDA, and the CUIs were words in the document. We used the lda-c software [68], which is a very efficient implementation of the LDA method, to conduct topic modeling.

When LDA is used in topic modeling for general documents (eg, news, scientific literature), words and their frequencies in the documents are used. However, in our analysis, we aimed to understand the medical concepts related to BII from social media texts. Different words may indicate the same medical concepts. For example, joint aches, painful joints, arthralgia, and aching joints all indicate joint pain and are associated with a single medical concept represented by a single CUI. Therefore, instead of using words, we used medical concepts, represented by CUIs, in our LDA analysis. Because multiple words indicating the same medical concept can be mapped to the same CUI, using CUIs can also aggregate and strengthen the information from multiple words, compared with using words, which may be sparse and thus not easy to learn topics from.

## Results

### cTAKES Annotations

Table 2 presents the summary statistics for the annotated mentions and their CUIs mapped by cTAKES. In BIIweb, cTAKES extracted 2186 mentions and mapped them to 475 unique CUIs. In HealingBII, cTAKES extracted 11,080 mentions and mapped them to 1177 unique CUIs. In the largest data set IG-BII, cTAKES extracted 5530 unique mentions and mapped them to 2871 unique CUIs. Note that the same mention can be mapped to multiple CUIs and can have multiple categories (each CUI has only one category). For example, the mention *flashes* is mapped to 2 different CUIs and then 2 different categories: diseases and medical procedures. Table 2 presents the statistics for each category of extracted mentions. For each data set, most of the extracted mentions were categorized as signs and symptoms by cTAKES.

**Table 2:** Statistical summary of annotations of the clinical Text Analysis and Knowledge Extraction System.

Data set	cwords^a^	annots^b^	maps^c^	M^d^	C^e^	M/C^f^	C/M^g^	S^h^	D^i^	P^j^
BIIweb	24,034	2186	661	640	475	1.39	1.03	385	149	106
HealingBII	163,352	11,080	1740	1685	1177	1.48	1.03	891	503	292
IG-BII	3,116,966	185,339	5694	5530	2871	1.98	1.03	3049	1549	932

^a^cwords: the total number of words recognized by the clinical Text Analysis and Knowledge Extraction System.

^b^annots: the total number of extracted mentions belonging to the 3 semantic types (ie, signs, symptoms, diseases, disorders, and medical procedures).

^c^maps: the number of unique mention–concept unique identifier mappings.

^d^M: the number of unique extracted mentions.

^e^C: the number of unique mapped concept unique identifiers.

^f^M/C: the average number of extracted mentions mapped to a given concept unique identifier.

^g^C/M: the average number of concept unique identifiers mapped to an extracted mention.

^h^S: the number of unique extracted mentions mapped to the signs and symptoms category.

^i^D: the number of unique extracted mentions that are mapped to the diseases and disorders category.

^j^P: the number of unique extracted mentions mapped to the medical procedures category.

To determine if cTAKES can sufficiently extract relevant mentions, we performed a manual annotation and compared the 2 lists of extracted mentions: one from using cTAKES and the other from using manual annotation. We randomly sampled 50 posts from each of the 3 data sets and manually annotated these posts. Upon manual annotation, we extracted mentions (words or phrases) that conveyed the concerns and experiences of social media users involving BII-related symptoms, diseases, and medical procedures. For a random sample of 50 posts (l_avg_=134.18) from BIIweb, we obtained a total of 575 mentions from using manual annotation, and 637 mentions using cTAKES; there were 479 common mentions. Each mention was associated with a post identifier and a character offset. A mention was considered to belong to both lists if it occurred in both lists with the same post identifier and character offset. We found that 83.3% (479/575) of manually annotated mentions were covered by cTAKES. This high coverage demonstrates that cTAKES can capture most of the relevant medical concepts. In contrast, 75.2% (479/637) of the annotated mentions by cTAKES were covered by manual annotation. This further demonstrates that most of the annotated mentions of cTAKES can be confirmed by manual annotation. Similarly, for a random sample of 50 posts (l_avg_=80.02) from HealingBII, 69.5% (194/279) of manually annotated mentions were covered by cTAKES; 70.3% (194/276) of mentions annotated by cTAKES were confirmed by manual annotation. For a random sample of 50 posts (l_avg_=121.00) from IG-BII, the corresponding values were 75.2% (182/242) and 64.3% (182/283), respectively. According to the high overlap in the results between manual annotation and cTAKES across multiple data sets used in our study, it is reasonable to assume that cTAKES is a decent surrogate of manual annotation for BII study through social media data.

### LDA Topics

To identify the best topic models, we used a grid search to identify the best parameter values for the Dirichlet prior α ∈ {0.01,0.05,0.1,0.5,1,1.5,2,5,10,15,20,25} and the number of topics K ∈ {3,4,5,10,15,20}. To evaluate topic models, we analyzed each LDA topic modeling result for every combination of α and K values corresponding to low perplexity scores [66,69,70].

For each topic modeling result, we analyzed the document-topic and topic-CUI probability distributions to derive topics and their respective top 10 representative mentions. The top 10 representative mentions for a given topic were the most frequent mentions, corresponding to the top 10 CUIs, with the highest probabilities of belonging to the topic. Multiple mentions could be mapped to a given CUI (Table 2). We only presented the most frequent mention because all mentions mapped to the same CUI had similar semantics. We further evaluated the quality of topic modeling based on how well the derived topics summarized the most representative mentions. We analyzed each LDA topic modeling result for every combination of α and K and chose the one where the derived topics were distinct and best summarized the most representative mentions. Finally, we identified distinct and meaningful topics using (1) K=4 and α=10 for BIIweb, (2) K=5 and α=10 for HealingBII, and (3) K=5 and α=1.5 for IG-BII. We observed that with higher K values, the most representative mentions were similar across the topics. Hence, the derived topics were not distinct and were difficult to interpret.

Tables 3-5 present the top 10 representative mentions, the frequencies of CUIs corresponding to the mentions (in %), and the interpretations of the topics indicated by the mentions (eg, common signs and symptoms). Note that the frequencies of CUIs are among all the posts, not only in those posts with the highest probability belonging to a certain topic. We presented these frequencies because each post had a certain probability of belonging to a certain topic, and thus frequencies among all posts would better represent the topic information across all the posts. These tables also present examples of posts that have a high probability of belonging to the respective topic. In the examples, the mentions that had high probabilities of belonging to the corresponding topics are italicized. Note that we used CUIs in LDA to derive the topic and word distributions (as discussed in the section Methods—Topic modeling), but we have presented the most frequent mentions (with clear semantics) that were mapped to the respective CUIs (which are identifiers without semantics) in these tables. The mentions in these tables were sorted based on the probabilities of their corresponding CUIs belonging to the respective topics. Please note that these probabilities have not been presented in the tables (they are not the frequencies presented in the tables). Therefore, each topic was represented by its most representative mentions, and thus, summarized such mentions. For example, we interpreted a topic as pain and other signs if there were a significant number of mentions related to pain, such as neck pain, chest pain, and headache. Please note that the topics have not been sorted, and the first columns in Tables 3 to 5 are nominal identifiers. Below, we have discussed the topics derived from LDA for BIIweb and HealingBII data sets from the original posts. Note that 2 topics can still share the same representative mention with different probabilities in the LDA.

**Table 3:** Derived topics in BIIweb.

Topic	Top 10 mentions	Interpretation
1	Testing (2.34); illness (4.46); problem (2.82); work (1.17); swollen (0.78); drains (0.61); feel common (2.51); fatigue (1.82); exhausted (0.39); sensitivity (0.95) Example: “I had silicone implants done 5 years ago, three years ago after going to the doctor with extreme *fatigue*^a^ (I was sleeping 14-16 hours a day and was still *exhausted*)”	Common signs and symptoms
2	Breast implant (6.80); removal (1.30); cancer (0.95); autoimmune (0.95); infection (0.87); scleroderma (0.39); pain (3.68); diagnosis (0.30); alcl (0.30); breast cancer (0.30) Example: “I had stage 4 breast *cancer* and had chemo and radiation. I tried to have my *breast implants* removed due to *pain*...Then I had an acute *infection* occur a month and a half after they put the new implants in and they were forced to perform an emergency *removal* of the newer implants. I have had all the symptoms of breast implant illness—even after their removal.”	Diseases or disorders
3	Breast implant (6.80); illness (4.46); toxicity (1.17); foreign body (0.87); heal (0.78); support (0.65); rupture (0.52); cancer (0.95); awareness (0.35); inflammation (0.56) Example: “...I never had a problem until 2006 at which time I thought something had happened however, my surgeon said I must have just pulled a muscle and that the *implants* seemed fine. Now that surgeon is old and the shop is closed up. I have been suffering for the past 13 years with arthritis, fatigue, brain fog, *inflammation*, hormone imbalances, and adrenal fatigue...”	Toxicity
4	Pain (3.68); feel (2.51); fatigue (1.82); back pain (0.87); illness (4.46); joint pain (0.56); worse (0.65); anxiety (0.52); ear ringing (0.39); headache (0.39) Example: “It wasn’t until 2017 where I started to experience *anxiety* and panic attacks (which I didn’t know I was having at the time). With that, along came crazy *headaches*, feeling dizzy, sick, lightheaded, and my right eye would always be swollen and never knew why.”	Pain and stress-related disorders

^a^The mentions in the examples that had high probabilities of belonging to the corresponding topics are italicized.

**Table 4:** Derived topics in HealingBII.

Topic	Top 10 mentions	Interpretation
1	Rupture (1.34); supported (0.87); read (1.17); suffering (0.87); happy (0.6); mastectomy (0.46); work (0.96); scare (0.77); reconstruction (0.41); mri (0.72) Example: “Double *mastectomy*^a^ in 2015. *Reconstruction* process with expanders then permanent 1000 ml saline implants in early 2016. After that was 9 procedures, a hysterectomy and now MANY health problems.”	Surgeries and procedures
2	Pain (3.91); joint pain (0.79); fatigued (0.96); ailment (4.70); removal (0.84); hair loss (0.52); headache (0.47); muscle ache (0.34); rash (0.39); infection (0.84) Example: “In addition to the neuromuscular spasms and *pain*, I’ve suffered with incapacitating chronic *fatigue*, BRAIN FOG and confusion (yes, even while driving), loss of vision and hearing, vertigo, mysterious skin *rashes, hair loss, migraines...*”	Pain and other signs
3	Problem (2.64); cancer (0.90); autoimmune (0.57); breast cancer (0.38); scars (0.35); treatment (0.43); diagnose (0.29); autoimmune disorder (0.27); lupus (0.29); arthritis (0.26) Example: “I had capsules form on both breasts from about 2010. I got sick with BII symptoms from 2005 with lots of infections required intravenous and oral antibiotics. My environmental and drug allergies got worse, onset of *arthritis*, skin rashes, *autoimmune* symptoms, started growing low grade *cancers...*”	Cancer and other disorders
4	Breast implant (3.85); ailment (4.70); toxicity (3.05); healing (1.56); capsulectomy (0.64); infection (0.84); inflammation (0.39); detoxification (0.32); foreign object (0.25); bleed (0.23) Example: “Some women with silicone *toxicity* have bruising and *bleeding* problems. If I was you, I would try and have the lymph node localized and checked for silicone and removed if it is contaminated beyond detoxing much like a silicone granuloma is removed.”	Toxicity
5	Emotion (3.70); think (2.26); feel (0.84); normal (0.65); anxiety (0.50); ill (0.61); sensation (0.33); tired (0.28); sores (0.27); depression (0.33) Example: “Even more heartbreaking and discouraging, has been the *emotional* pain of not being able to freely play with her on the floor due to hip and knee pain, along with leg and foot spasms...but I struggle with many *feelings* of failure as a wife and mother due to physical limitations.”	Mental health

^a^Italic text indicates the mentions in the examples that had high probability of belonging to the corresponding topics.

**Table 5:** Derived topics in IG-BII.

Topic	Top 10 mentions	Interpretation
1	Heal (1.46); working (0.90); weighted (1.05); able (0.99); rest (0.37); stress (0.29); exercise (0.28); therapeutic (0.35); sleep (0.36); run (0.23) Example: “It’s been 14 months since my explant. The journey to *healing*^a^ hasn’t been an easy one due to setbacks and relapses but better than daily anaphylaxis from getting cold, food, smells, crying, *exercise* and *stress*, then add angina attacks from anaphylaxis.”	Physical health
2	Malignancy (1.10); removal (0.96); scar (0.75); capsulectomy (0.68); rupture (0.43); ciactrice (0.43); alcl (0.41); augmentation (0.37); lymphoma (0.35); removal of implants (0.29) Example: “The ugly side of breast implants. It’s not a matter of IF you will get sick...it’s WHEN. implants leak toxic heavy metals without rupture It’s called a gel bleed. Women with implants are 3 times more likely to develop brain, lung and *lymphatic cancer* than women with implants.”	Cancer and medical procedures
3	Loving (2.43); happiness (2.11); emotion (1.64); think (1.05); feel (0.87); scare (0.55); confidence (0.35); tired (0.38); emotional (0.27); sensation (0.33) Example: “I was *scared* of looking incomplete. After much deep, inner work on myself, I realized that my worth wasn’t dependent on what I looked like or how big my chest was. I realized that true *happiness* would come from 100% acceptance of what and who I was”	Mental health
4	Breast implant (7.21); ailment (5.67); toxicity (1.67); aware (0.96); felt worse (0.36); test (0.64); foreign body (0.45); alone (0.33); suffering (0.21); complication (0.20) Example: “...We get *toxic* from the chemical makeup of the silicone, the *toxic* chemicals that are released when the shell degrades, sick from rupture and sometimes mold.”	Toxicity
5	Pain (2.52); inflammatory reaction (0.89); fatigue (0.83); anxiousness (0.72); allergy (0.43); depression (0.37); joint pain (0.33); autoimmune disorder (0.32); swell (0.43); infection (0.31) Example: “For three years, doctors have been unable to diagnose or explain upper body weakness, hand *pain*, and general *inflammation*. I have suffered from periods of high *inflammation*, debilitating *fatigue*, migraines, inability to lose weight, insomnia, low libido, body and *joint pain*, hair loss, dry skin, dry eyes, brain fog, etc.”	Common disorders

^a^Italic text indicates the mentions in the examples that had high probability of belonging to the corresponding topics.

Table 3 presents the topics in the data set BIIweb data set. Although BIIweb was the smallest the data set (Table 1), we were still able to identify 4 distinct topics with the most representative mentions, namely, fatigue, infection, toxicity, and anxiety. Table 4 presents the topics in the data set HealingBII, which shared some common topics and representative mentions with those in BIIweb. For example, pain, cancer, and toxicity were common across these 2 data sets. However, a focused topic unique to HealingBII was surgeries and procedures, where people (mostly patients) discuss the procedures among themselves and share their related experiences. Another unique topic in HealingBII was mental health.

In addition to physical symptoms, individuals reported significant emotional and mental difficulties, such as depression, and expressed serious symptoms on social media. Table 5 presents the topics in the data set IG-BII data set. IG-BII was the largest data set (Table 1) and had significantly more posts than the other two. We observed that cancers, mental health, and toxicity emerged as significant topics in this large data set, consistent with those in HealingBII. In IG-BII, people also discussed their recovery process from the issues or events associated with BII. We identified from these 3 data sets frequent mentions of rupture, pains, and fatigue. We also identified mentions of cancer, lupus, and autoimmune disorders. Please note that Table 3 contains 4 topics for BIIweb, but Tables 4 and 5 contain 5 topics for HealingBII and IG-BII, respectively. This is because the number of topics was determined by how distinct the topics were, not by the prespecified number of topics.

Table 6 presents the top 10 representative mentions, the frequencies of CUIs corresponding to the mentions (in %), and interpretations of the topics on the unified data set, combining all 3 data sets BIIweb, HealingBII, and IG-BII. We obtained a unified data set by combining all the posts from the 3 data sets into one corpus. To perform topic modeling, we processed the posts in the unified data set in the same way as we processed the posts in the individual data sets (discussed in the section Methods—Topic modeling). Upon topic modeling, we identified 5 distinct topics using K=5 and α=1.5. We observed that physical health, cancers, mental health, toxicity, and common disorders emerged as significant topics in the unified data set, consistent with those in IG-BII. This was because IG-BII was the largest data set out of the three and comprised 93.22% (28,987/31,094) of the unified data set. We also identified common concerns such as pain, allergy, depression, weight gain, cancer, inflammation, and toxicity issues from the individual and unified data sets. This implies that the above-mentioned factors were frequently associated with BII.

**Table 6:** Derived topics in the unified data set.

Topic	Top 10 mentions	Interpretation
1	Working (1.45); ate (0.92); weight (0.79); runs (0.40); thinking (2.68); exercise (0.25); talk (0.50); walking (0.35); nutrition (0.15); move (0.28); Example: “...I’m now healthier than I have been in the last 7 years of my life!...I explanted in Feb of 2018, a few months after explant, I gained my *weight*^a^ back and found a love for true self care and *working* out.”	Physical health
2	Illnesses (4.45); cancer (0.87); ruptures (0.77); removal (0.76); awareness (0.73); suffers (0.83); capsulectomy (0.54); autoimmune (0.52); breast augmentation (0.30); augmentation (0.28); Example: “I was diagnosed with breast *cancer* at the young age of 30 and ended up with a double mastectomy as part of that process...now 10 years later I have just 15 weeks ago had my implants removed. They had *ruptured*, were toxic and giving me health issues”	Cancer and medical procedures
3	Feel (5.94); loved (2.97); thinking (2.68); happier (1.64); feelings (1.47); afraid (0.66); confidence (0.27); support (0.79); able (0.77); alive (0.17); Example: “When I found out I was sick and I had to tear apart my body to get better I never thought I’d be happy with myself again. I am 4 weeks post op and *feeling* more happy and healthy than ever. I was worried I’d never be *loved* again.”	Mental health
4	Heal (2.26); scars (0.58); scarred (0.33); drain (0.26); toxic (1.97); sights (1.25); inflammation (0.68); bulge (0.36); tenderness (0.20); red (0.15); damage (0.16); Example: “I was so worried about how *red* and raised up my *scars* were...then they got really inflamed, sore and raised up around 3 weeks and i was really stressed over it. then overnight the *inflammation* and redness went down...”	Common signs, symptoms, and toxicity
5	Pain (2.09); tired all the time (0.69); anxiety (0.57); joint pain (0.46); alopecia (0.39); weight gain (0.37); allergies (0.35); depression (0.29); pain back (0.23); headache (0.22) Example: “Before I had the explant, I had many unexplained symptoms (brain fog, *joint pain*, back and neck pain, *tired all the time*, psoriasis, afib, just to mention a few) since I awoke from surgery I have had absolutely no neck, back, or joint pain.”	Common disorders

^a^Italic text indicates the mentions in the examples that had high probability of belonging to the corresponding topics.

Table 7 presents the percentage of posts per topic, where a post *d* is considered to belong to a topic z if among all topics that *d* has, z has the highest probability. Although the distributions are not completely consistent across data sets, toxicity remained a notable topic among all data sets. This indicates that these were common issues that were significantly associated with BII. In addition, pain, cancer, mental health, and other disorders were also associated with breast implants.

**Table 7:** Distribution of posts among the topics.

Data set and topics		Posts, n (%)
**BIIweb**		
	Common signs and symptoms	62 (33.2)
	Diseases or disorders	28 (15)
	Toxicity	50 (26.7)
	Pain and stress-related disorders	47 (25.1)
**HealingBII**		
	Surgeries and procedures	713 (37.1)
	Pain and other signs	221 (11.5)
	Cancer and other disorders	221 (11.5)
	Toxicity	505 (26.3)
	Mental health	260 (13.6)
**IG-BII**		
	Physical health	11,299 (39)
	Cancer and medical procedures	3890 (13.4)
	Mental health	4879 (16.8)
	Toxicity	5415 (18.7)
	Common disorders	3504 (12.1)
**Unified**		
	Physical health	4760 (15.3)
	Cancer and medical procedures	10,637 (34.2)
	Mental health	7954 (25.6)
	Common signs, symptoms, and toxicity	4030 (13)
	Common disorders	3713 (11.9)

## Discussion

### Principal Findings

To understand the signs, symptoms, and diseases or disorders associated with BII, a condition reported primarily on social media rather than in medical reports, we collected social media posts and analyzed them using NLP and topic modeling. We extracted mentions related to signs, symptoms, diseases, disorders, and medical procedures using cTAKES, mapped them to standard medical concepts, and summarized the mapped concepts to topics using LDA. We found that mentions such as rupture, infection, inflammation, pain, and fatigue were common self-reported issues. We also found that mental health–related concerns such as stress, anxiety, and depression, as well as diseases such as cancers and autoimmune disorders, were common concerns. The cTAKES was able to extract medication and anatomy information as well, but they were not used in our LDA analysis, given that the objective of our study was not to study the medications used or the anatomy related to BII.

In our method, we relied on cTAKES and the rich UMLS dictionary to extract all relevant mentions, including their lexical variants (synonyms, abbreviations, paraphrases). To determine if cTAKES could sufficiently extract relevant mentions, we performed a manual annotation to extract all the relevant mentions and compared them with the extracted mentions from cTAKES. We found that cTAKES could sufficiently capture relevant medical concepts and was comparable with manual annotation. It is worth noting that we did not evaluate the performance of our mention extraction module on all the posts of each data set, which is typically performed using precision and recall metrics when there are ground-truth labels associated with each mention. However, in order to have such labels, careful manual annotations based on domain knowledge of BII are required. Unfortunately, such domain knowledge on complications, symptoms, and other issues associated with or caused by BII were not fully available. Our goal in this study is to provide useful information from social media data that could complement our current knowledge. Therefore, in this preliminary study, we used all annotated mentions, assuming that cTAKES enabled high-quality annotations.

### Strengths and Limitations

We acknowledge that cTAKES might not have been able to extract all relevant mentions from our social media data sets. This is because cTAKES was originally designed for extraction of medical entities from clinical notes, which have very different wording and writing styles compared with social media data. As social media data comprise informal phrases, short ambiguous texts, emoticons, and a wide range of lexical variants corresponding to a single concept, cTAKES might not work flawlessly on social media data, although we observed reasonable output from cTAKES. We also observed that cTAKES often associated a single mention with multiple CUIs belonging to the same category. We think this was because of the presence of multiple mappings for a given mention in the UMLS metathesaurus. Regardless, the extracted mentions and the mapping of mentions to UMLS CUIs, as generated by cTAKES, were used for topic modeling without any manual verification or evaluation. In the future, we will develop a detailed guideline to further evaluate the extracted mentions before using them in topic modeling.

Our study had some limitations. First, LDA is an unsupervised learning technique in which the number of topics (K) is assumed to be known a priori. However, it is difficult to accurately estimate K for a given data set. In our study, we used a grid search to obtain different K values. Even without full domain knowledge, it remains nontrivial to evaluate the LDA results for each K value. In our study, we selected the topics based on α and K values. We did not use perplexity [66,69,70], a widely used metric in topic modeling, to select the topics, because as studied in the literature (eg, Chang et al [71]), perplexity often does not correlate well with topic interpretability; in our case, the lowest perplexity did not always enable intuitive or meaningful topics. In the future, we will develop more rigorous ways to select the number of topics and evaluate the topic modeling results. In this study, we did not conduct a sentiment analysis of the posts to understand the positive or negative opinions expressed in the posts. We plan to include this process before topic modeling to generate a cleaner data set for topic modeling.

It is worth noting that social media data could be of variable quality (eg, misspelling, misconception, and biased opinions), particularly compared with medical literature data. Anyone can post on social media, and so the derived content may be from individuals who may have other implant-specific issues such as capsular contracture or implant infection. Thus, understanding the diseases, disorders, symptoms, signs, etc, associated with a drug, disease, or medical procedure from social media data would always be at risk from confounders or errors. However, given that the medical knowledge and literature on BII have not been well established, and the related concepts are not well defined or well accepted, using social media data to understand emerging issues could be a meaningful starting point. Still, any findings from social media data would require a rigorous evaluation and validation based on medical and biological knowledge, experiments, clinical practice, etc. In addition, we have only analyzed 3, though the most relevant and prolific websites dedicated to BII discussions. A more comprehensive analysis of social media data on a much larger scale would be beneficial to better understand BII in a larger, diverse population. Sentiment analysis of social media data could be another valuable analysis to enable more insights into the health experiences of users or patients and their emotions or feelings. We will consider sentiment analysis in our future research when BII is better understood, and we can accurately annotate social media data.

### Conclusions

This study has important implications for future methodological and clinical research. Future methodological research on NLP could include causality inference between BII and symptom and sign mentions from social media to understand their relations, etc. Our findings could provide the relevant domains for clinical research studies seeking to develop measures of BII and to identify its causes. More specifically, our results can provide a patient-derived definition of BII, which can be useful to clinicians treating patients with BII concerns to use this patient-centered language. Our methods and informatics strategies applied in this study would also provide working examples for analyzing other emerging but not well-defined illnesses from social media data.

Our analysis of social media data identified mentions such as rupture, infection, inflammation, pain, and fatigue, which were common self-reported issues on social media sites dedicated to BII. In addition, our analysis showed that a significant number of user comments and posts were also concerned with mental and physical health and toxicity issues after having breast implants. The findings from our study could be used to further the scientific study of BII, as well as the care of patients presenting with the described symptoms, by allowing clinicians to develop a patient-centered language to better approach the patients with concerns. Our study provides the first analysis and derived knowledge of BII from social media using NLP techniques and demonstrates the potential of using social media information to better understand emerging illnesses.

## Appendix

Multimedia Appendix 1 A brief description of latent Dirichlet allocation.

## Abbreviations

ASCII: American Standard Code for Information Interchange
BII: breast implant illness
cTAKES: clinical Text Analysis and Knowledge Extraction System
CUI: concept unique identifier
LDA: latent Dirichlet allocation
NLP: natural language processing
UMLS: unified medical language system
XMI: XML metadata interchange
