# Supplementary material for: A Pipeline to Understand Emerging Illness Via Social Media Data Analysis: Case Study on Breast Implant Illness
Source: JMIR Med Inform. 2021 Nov 29;9(11):e29768. doi: 10.2196/29768 (PMC8669576; doi:10.2196/29768)
Supplement: Multimedia Appendix 1 [file medinform_v9i11e29768_app1.docx]

**Original Paper**

**Authors:**

Vishal Dey, BS^1^

Peter Krasniak, MD^2^

Minh Nguyen, MD^2^

Clara Lee, MD^2^

Xia Ning, PhD^1,3, 4^

**Affiliations:**

1. Computer Science and Engineering, The Ohio State University, Columbus, OH

2. Plastic and Reconstructive Surgery, The Ohio State University, Columbus, OH

3. Biomedical Informatics, The Ohio State University, Columbus, OH

4. Translational Data Analytics Institute, The Ohio State University, Columbus, OH

*Corresponding Author

Xia Ning

1800 Cannon Drive, 310C, Columbus, OH 43210

[ning.104@osu.edu](mailto:ning.104@osu.edu)

614-366-2298

**A Pipeline to Understand Emerging Illness via Social Media Data Analysis: A Case Study on Breast Implant Illness**

**Brief Description of LDA:**

**
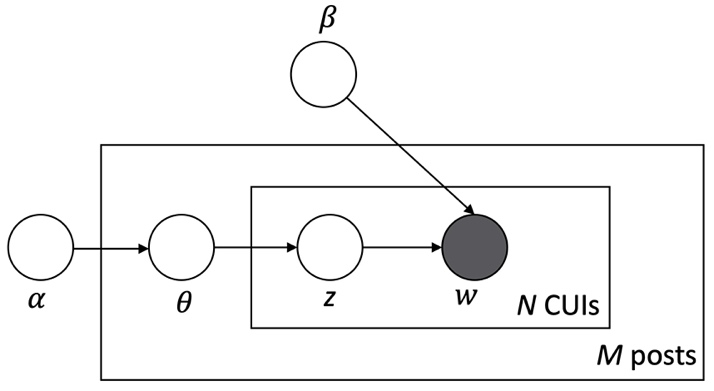
**

Figure S1: Graphical model of LDA

Here, we briefly describe Latent Dirichlet Allocation (LDA).[1] LDA is a generative probabilistic model that discovers latent topics in a document corpus. LDA assumes that a document of $N$ words $\boldsymbol{w}=\{w_{1}, w_{2},\cdots,w_{N}\}$ is generated as follows: 1) a per-document distribution over topics $\boldsymbol{\theta}\in\mathbb{R}^{K}$ is first generated from a Dirichlet distribution Dirichlet($\boldsymbol{\alpha}$), where $\boldsymbol{\alpha}\in\mathbb{R}^{K}$ is the Dirichlet prior $\alpha_{k}\geq0$ $(k=1, \cdots,K)$ and $K$ is the given number of topics; 2) for each word $w_{i}$ in the document, a topic $z_{i}$ is generated from a multinomial distribution Mult($\boldsymbol{\theta}$); 3) a word distribution $\boldsymbol{\varphi}_{i}\in\mathbb{R}^{L}$ over topic $z_{i}$ is generated from a Dirichlet distribution Dirichlet($\boldsymbol{\beta}$), where $\boldsymbol{\beta}\in\mathbb{R}^{L}$ is the Dirichlet prior, $\beta_{l}\geq0$ $(l=1, \cdots,L)$ and $L$ is the number of words in the vocabulary; 4) given $\boldsymbol{\varphi}_{i}$, word $w_{i}$ is generated from a multinomial distribution Multi($\boldsymbol{\varphi}_{i}$). LDA assumes all the words $w_{i}$ in a document are independent given their $\boldsymbol{\varphi}_{i}$, and all the documents in the corpus are independent. Estimation on $\boldsymbol{\theta}$ and $\boldsymbol{\varphi}$ via maximum likelihood methods will enable document topics and the most probable words over the topics.

**References**

1. Blei DM, Ng AY, Jordan MI. Latent Dirichlet allocation. Journal of Machine Learning Research. 2003;3:993–1022. doi: 10.1016/b978-0-12-411519-4.00006-9
